# Research on plasma modified fly ash denitration

**DOI:** 10.1038/s41598-024-54948-3

**Published:** 2024-02-22

**Authors:** Zhan-feng Qi, Shuo Wang, Xiu-li Guo

**Affiliations:** College of Mechanical Engineering, Dalian University, Dalian, 116622 China

**Keywords:** Fly ash, Plasma, Denitration mechanism, Characterization analysis, Environmental sciences, Materials science, Nanoscience and technology

## Abstract

The effects of reactor parameters and process parameters on the denitration rate of modified fly ash in different gas atmospheres were studied by using a dielectric barrier plasma reactor and using orthogonal experiments. The characteristics of modified fly ash were analyzed using scanning electron microscope, specific surface area analyzer, X-ray diffraction, Boehm titration and Fourier transform infrared spectroscopy. The experimental data were processed by variance analysis and linear regression to induce the denitration mechanism. *R*^2^ of the linear regression analysis model is 0.789, which means that the adsorption pore size, acid groups and basic group can explain 78.9% of the change in denitration rate. The basic group will have a significant positive impact on the denitration rate, and the adsorption pore size and acidic group will have a significant negative impact on the denitration rate. Through variance analysis of the experimental data, it was found that the input power and discharge gap have a significant effect on the denitration rate, but the ionization time and discharge length have no significant effect. The input power affects the denitration rate by impacting the basic group, and the discharge gap affects the denitration rate by influencing the adsorption pore size. There are three denitration mechanisms on the surface of fly ash: physical adsorption, chemical adsorption and absorption process. Among them, chemical adsorption is the main mechanism of action, accounting for approximately 60.86%.

## Introduction

The environmental issues caused by NO_x_ are a major safety hazard for social sustainable development. The global industrialization process has led to a significant increase in NO_x_ emissions, with automobiles being the main contributors to NO_x_ emissions and diesel vehicles accounting for 80% of the total emissions from automobiles^1^. Selective Catalytic Reduction (SCR) is one of the methods used to address NO_x_ emissions from diesel vehicles. However, as national standards for engine exhaust emissions become increasingly stringent, the requirements for catalyst performance have shifted to cost pressures. Expensive catalysts are not only a matter of raw materials but also a matter of complex preparation processes and energy consumption^2^. Therefore, the development of simple, effective, and energy-efficient methods for the preparation of high-performance catalysts is a research focus for scholars both domestically and internationally.

The resource utilization of solid waste fly ash (FA) for the preparation of denitration catalysts has been extensively researched by numerous experts and scholars in recent years^3^. Generally, FA is used alone as a carrier or combined with other materials to prepare denitration carriers. Lei et al.^4^ prepared denitration catalysts by mixing FA and bentonite in a certain proportion as raw materials. Duan et al.^5^ synthesized Mn0.15Fe0.05/FA catalysts and obtained high catalytic activity. Liu et al.^6^ synthesized catalysts by loading Mn–Ce oxide on pretreated FA, with a working temperature range of 200–300 °C. Zhang et al.^7^ utilized low-temperature plasma calcination technology to load heavy metal oxides onto fly ash-based catalysts in order to enhance their sulfur resistance. Despite the vast amount of research focusing on the role of fly ash as a denitration catalyst, the majority has involved the modification of catalysts by loading other components onto fly ash. There is still a lack of research on using fly ash itself as a catalyst and on the denitration of fly ash itself.

The methods of modifying fly ash materials can be divided into physical modification, chemical modification, and combined modification. However, these methods have certain limitations in terms of the utilization of fly ash resources and denitration treatment^8^. Fly ash contains a large amount of Si oxides, Al oxides, and Fe oxides, which can be activated by plasma modification. Sha et al.^9^ have used plasma reactors to modify fly ash and bentonite mixtures, demonstrating a certain denitration capabilities, with oxygen modification being the most effective. Sun et al.^10^ used plasma technology to modify fly ash and to remove HgO and achieved good results. However, these studies either did not consider the inherent properties of fly ash or did not specifically focus on denitration, and lacked attention to the mechanisms and parameters of the plasma reaction process.

Dielectric barrier discharge (DBD) plasma technology is a gas discharge technology that inserts insulating media into the discharge space. In this study, a DBD plasma reactor was used to investigate the effects of process parameters and reactor parameters on the denitration performance of fly ash through orthogonal experiments. The surface morphology, bulk phase, surface acidity, and their relationship with catalytic activity of modified fly ash were studied using scanning electron microscopy (SEM), BET specific surface area analyzer, X-ray diffraction (XRD), X-ray Fluorescence Spectroscopy (XRF), Boehm titration, and Fourier transform infrared spectroscopy (FTIR). Furthermore, the mechanism of plasma modification on the denitration of fly ash was analyzed.

## Materials and methods

**Figure 1:**
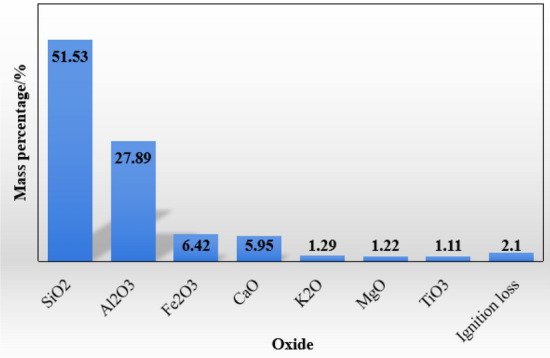
Composition and content of oxide in fly ash.

Fly ash was used as the catalyst in this experiment. fly ash was obtained from Dalian Huaneng Power Plant, and its composition proportions are shown in Fig. 1.

### Experimental procedure

**Figure 2:**
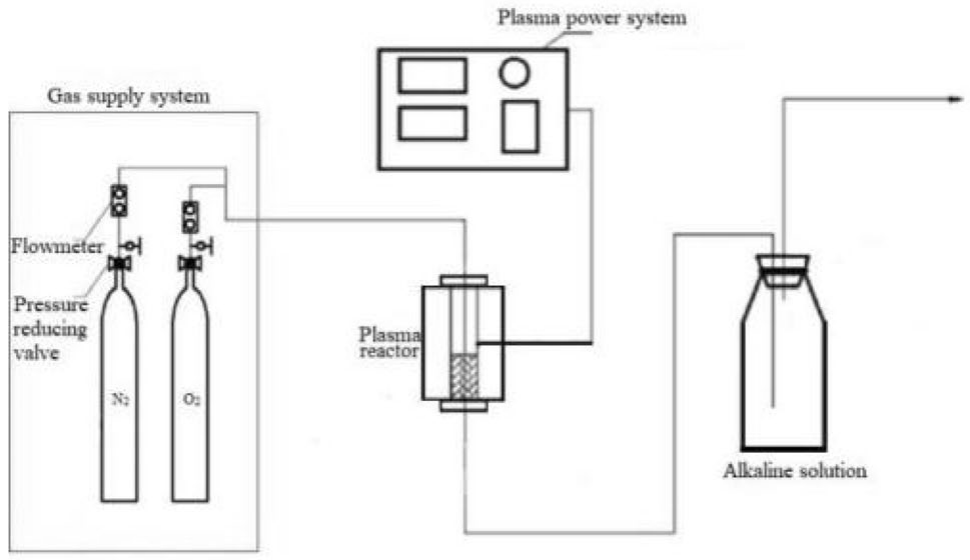
Schematic diagram of the plasma-modified system.

1. *Modification Process* The plasma modification system is shown in Fig. 2 and consists of a high-voltage pulse power supply, plasma reactor, gas supply system, and alkaline absorption solution. The gas supply system is composed of N_2_ and O_2_ gas cylinders, which control the airspeed of the reaction system through pressure reducing valves and flowmeters. The plasma reactor adopts a coaxial cylindrical double dielectric layer structure composed of a high-voltage electrode (copper rod), ground electrode (iron wire mesh), and discharge medium (quartz tube). During the experiment, the gas supply system controls the gas changes, and the input power and ionization time are adjusted by the high-voltage pulse power supply (CTP-2000). The length of outer iron wire mesh is changed to control the discharge length, and the diameter of inner copper tube is changed to control the discharge gap.

Fly ash was washed with deionized water, dried, ground, and sieved through a 200-mesh screen. 5 g of fly ash was evenly spread on the discharge area using a spoon at a time. The output power range of the high-voltage pulse power supply was between 1 and 80 W, and the output frequency was between 1 and 30 kHz.

**Figure 3:**
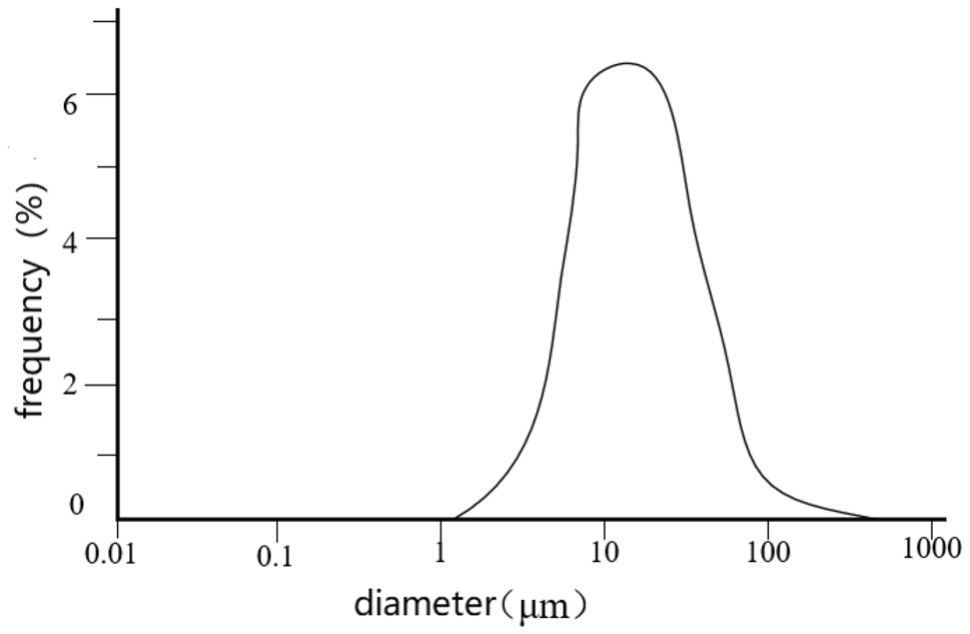
Particle size distribution of original fly ash.

The refractive index of fly ash is set to 1.54, with water as the dispersing medium having a refractive index of 1.333. The original particle size distribution of the ash is measured using a laser particle size analyzer as shown in Fig. 3.

**Figure 4:**
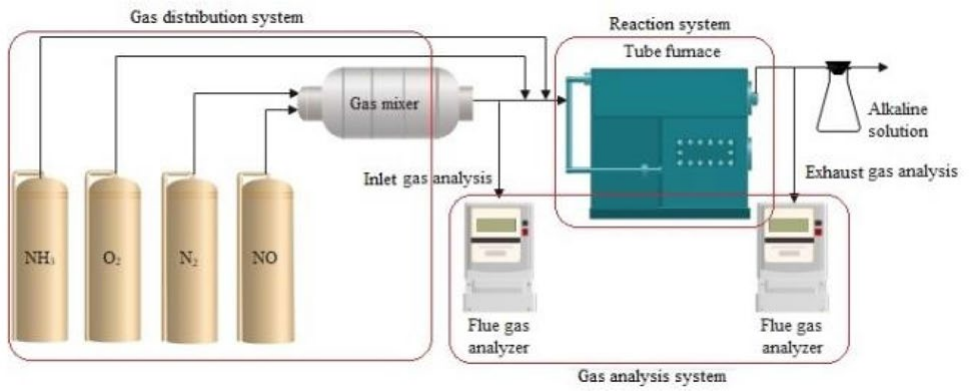
Schematic diagram of the denitration evaluation device.

(2) *Denitration Evaluation Process* The catalyst activity evaluation system includes a dynamic gas supply system, reaction system, and gas analysis system, as shown in Fig. 4. The reaction system is composed of a quartz tube and a tubular furnace, and the reaction temperature is controlled by adjusting the tubular furnace heating temperature. The dynamic gas supply system is composed of gas cylinders and flow meters pressure reducing valves, while the gas analysis system detects the inlet and outlet gases with a flue gas analyzer.

The gas composition of the dynamic gas supply system is as follows: the NO concentration is 0.1%, the O_2_ concentration is 5%, the NH_3_ concentration is 1%, N_2_ is used as the balance gas, and the gas input airspeed is 15,000 h^−1^. To prevent NO from reacting with O_2_ before entering the tubular furnace, O_2_ is directly introduced into the tubular furnace, like NH_3_. The activity evaluation temperature is 350 °C.

### Performance evaluation

As the core of the SCR system, the catalyst’s function is to eliminate NO_x_ from the exhaust gas, and the denitration rate directly reflects the effect of NO_x_ removal.

1 $$ Denitration\;rate\left( \% \right) = \frac{{\left[ {NO_{x} ]_{in} - } \right[NO_{x} ]_{out} }}{{[NO_{x} ]_{in} }} \times 100\% $$

where [NO_x_]_in_ is the inlet NO_x_ concentration, and [NO_x_]_out_ is the outlet NO_x_ concentration.

### Characterization

SEM analysis was performed using a Korean company’s EM-30 plus scanning electron microscope to observe the surface morphology of fly ash material. The specific surface area analysis was carried out using a Micromeritics ASAP 2010 rapid surface area and pore size analyzer, and calculated based on the BET equation. The surface functional group content of fly ash was determined using the Boehm method. The composition and elemental content changes of the original ash and modified fly ash were determined using X-ray fluorescence spectroscopy (XRF) analysis.For the catalyst crystal structure, testing was conducted using the X-ray diffractometer (XRD-6100). The testing range was from 20° to 80°, with a scanning speed of 16°/min, Cu-Kα radiation source, 40 kV tube voltage, and 40 mA tube current. Fourier transform infrared spectroscopy was used to determine the infrared spectra of the catalyst surface using the Spectrum 400. The spectral information was recorded in the wavelength range of 4000–500 cm^−1^.

## Results and analysis

### Orthogonal experimental design

Plasma modified denitration is a reaction influenced by multiple factors. Adopting the orthogonal experimental method and variance analysis of experimental data can not only help in finding the best combination of factor levels with a small number of experiments but also in obtaining the main and secondary relationships and interaction effects between various factors. Studies^11–13^ have shown that, among the reactor parameters, the discharge gap and discharge length have a significant effect, while among the process parameters, the input power and ionization time have a significant effect. In addition, different modified gases also have different effects on the removal of nitrogen oxides. Therefore, the modified gas type was determined to be O_2_ and N_2_, and then 4 factors were selected under O_2_ atmosphere, each with 3 different levels, randomly arranged, as shown in Table 1.

**Table 1:** Factors and levels.

Level	Input power (W)	Ionization time (min)	Discharge length (mm)	Discharge gap (mm)
1	20	10	80	1
2	40	20	120	1.5
3	60	30	160	2

According to Table 1, 10 orthogonal experiments were conducted with denitration rate as the evaluation index. The results are shown in Table 2.

**Table 2:** Orthogonal test results.

No	Input power (W)	Ionization time (min)	Discharge length (mm)	Discharge gap (mm)	Denitration rate (%)
1	20	20	120	1.5	47.03
2	60	30	160	1.5	52.18
3	60	20	80	1	50.25
4	40	30	120	1	49.81
5	20	30	80	2	51.23
6	40	10	80	1.5	53.61
7	40	20	160	2	53.23
8	60	10	120	2	51.14
9	20	10	160	1	38.88
10	20	10	80	1	42.83

Variance analysis was performed using Multilevel Categoric analysis in Design-Expert13, and the results are shown in Table 3.

**Table 3:** Analysis of variance.

	Sum of squares	d*f*	Mean square	*F*	*p*
Model	205.33	8	25.67	419.04	0.0378*
Input power	80.75	2	40.37	659.16	0.0275*
Ionization time	17.46	2	8.73	142.57	0.0591
Discharge length	23.11	2	11.55	188.64	0.0514
Discharge gap	58.25	2	29.13	475.53	0.0324*
Residual	0.0613	1	0.0613		

*p < 0.05.

The order of the four factors affecting denitration rate is as follows: input power > discharge gap > discharge length > ionization time. The *p*-values indicate that input power and discharge gap have a significant impact on denitration rate, while the effects of ionization time and discharge length are not significant. The optimal parameter combination is an input power of 40 W, ionization time of 30 min, discharge length of 80 mm, and discharge gap of 2 mm. As this combination was not present in the experimental table, it was validated experimentally and yielded a denitration rate of 58.10%,which was higher than other combinations of factor levels.

### Plasma modification and denitration study

#### Gas modification study

**Figure 5:**
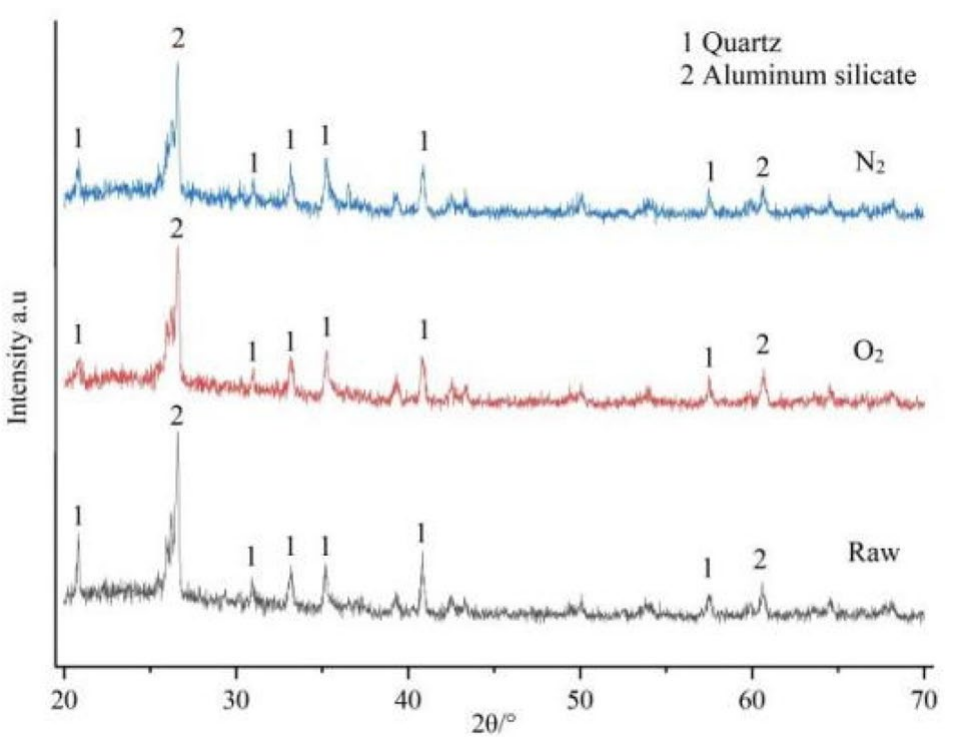
XRD of fly ash before and after modification with different gases.

Due to the introduction of gas during the modification process, the mineral composition of fly ash before and after oxygen gas modification was analyzed using XRD, as shown in Fig. 5.

As shown in the figure, fly ash is mainly composed of mullite (Al_6_Si_2_O_13_) and quartz (SiO_2_). The characteristic peaks of modified fly ash are similar to those of original fly ash, with some characteristic peaks of quartz weakened, indicating that modification has some effect on the crystal structure of fly ash, but there is no evidence to suggest the introduction of new elements in the crystal structure. This may also be due to the fact that the amount of substances produced by plasma modification is insufficient for detection by XRD analyzer.

This study utilized X-ray fluorescence (XRF) technology to determine the mineral composition and elemental content changes of fly ash in its original state and after oxygen modification, aiming to ascertain the effect of modification on the content of major components, and the results are shown in Tables 4 and 5. Table 4 presents a comparison of the elemental content changes of fly ash in its original state and after modification, while Table 5 compares the changes in the oxide content of fly ash in its original state and after modification. The oxide percentage content obtained in this experiment was derived from the elemental percentage content.

**Table 4:** Comparison of element content changes.

	O	Si	Fe	Al	Ca	K	Mg
Raw ash (%)	64.29	14.37	0.74	11.70	3.28	0.86	0.74
Modified (%)	63.74	13.69	2.10	10.91	4.16	0.84	0.51

**Table 5:** Comparison of composition changes.

	SiO_2_	FeO_x_	Al_2_O_3_	CaO	K_2_O	MgO	TiO_2_	SO_3_
Raw ash (%)	51.53	6.42	27.89	5.95	1.29	1.22	1.11	0.64
Modified (%)	50.50	8.60	26.03	7.22	1.22	0.90	1.30	0.67

From Table 4, it can be seen that after plasma discharge modification, there is no significant change in the content of each element in fly ash compared to before modification. Looking at the composition content changes of fly ash before and after modification in Table 5, it can be observed that the proportion of SiO_2_ and Al_2_O_3_ is the highest, corresponding to the major components mullite and quartz in fly ash, and there was no significant change in the composition proportions before and after modification. Tables 4 and 5 indicate that the plasma discharge method did not overly alter the content of elements and components in fly ash. The slight variations in the content of each component may be due to inadvertent errors caused by the non-uniformity of the selected samples and differences in the test area during the testing process.

**Figure 6:**
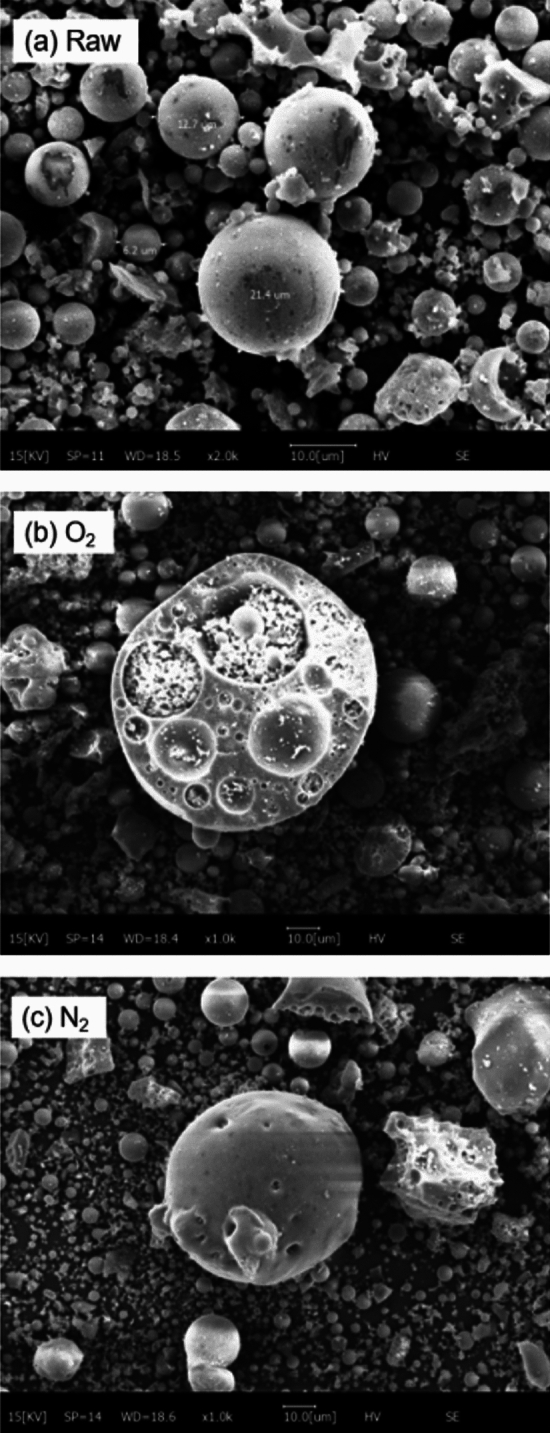
SEM analysis of fly ash.

**Figure 7:**
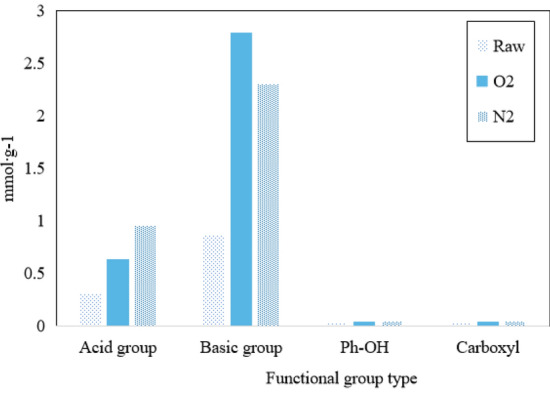
Titration results of fly ash.

**Figure 8:**
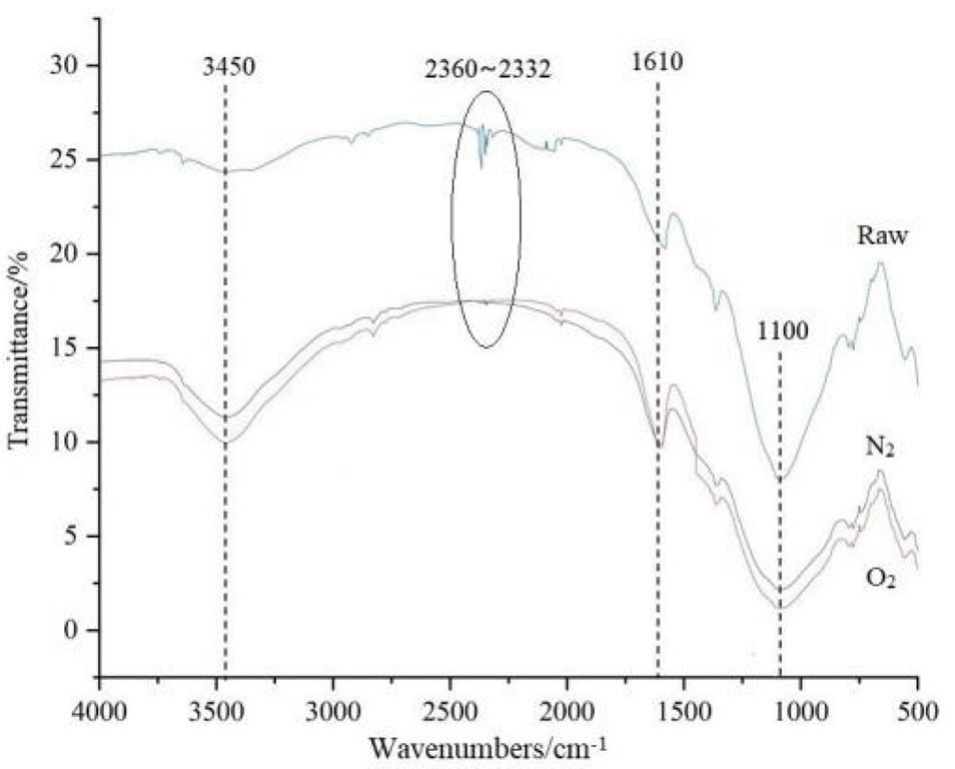
Infrared spectrum of fly ash.

The effects of gas atmosphere changes on fly ash were characterized by SEM, Boehm titration, and FTIR, as shown in Figs. 6, 7 and 8, respectively.

In Fig. 6, the untreated fly ash was mostly spherical particles of varying sizes, with a relatively regular shape. The morphology of modified fly ash changed significantly, with more small pores on the surface compared to the original fly ash, and an increased number of small particles. This was due to the destruction of the original structure of fly ash, exposing many small particles. Comparing Fig. 6b, c, it could be seen that the shell of fly ash was etched more during oxygen gas modification, increasing the surface area and reducing the adsorption pore size, which further improved the denitration rate.

The catalyst surface has four types of functional groups: acidic groups, basic group, phenolic hydroxyl groups, and carboxyl groups. In Fig. 7, unmodified fly ash surfaces have many basic group. After modification, both acidic and basic group increased by about 2–3 times. Phenolic hydroxyl and carboxyl groups are present in small quantities and do not change significantly after modification. Comparing with the same functional groups, O_2_ modification results in a more significant increase in basic group, while N_2_ modification results in a more significant increase in acidic groups. Based on the experimental results, it can be concluded that the denitration effect of O_2_ modification is better than that of N_2_, indicating that basic group have a more positive effect on improving denitration rate.

In Fig. 8, the composition and quantity of functional groups on fly ash surface before and after modification have changed significantly, with peaks at 3450 cm^−1^, 2360–2332 cm^−1^, 1610 cm^−1^, and 1100 cm^−1^ showing significant changes. The peak of 3450 cm^−1^ corresponds to the characteristic peak of O–H, 2360–2332 cm^−1^ corresponds to the characteristic peaks of C≡C and C≡N, 1610 cm^−1^ corresponds to the characteristic peaks of C=O, C=N, and N=O, and 1100 cm^−1^ corresponds to the characteristic peak of –O–.

Due to the drying process, the possibility of moisture content in fly ash is very low. The 3450 cm^−1^ peak corresponds to the O–H stretching vibration of fly ash surface, and its broadening and flattening indicates an increase in oxygen radicals. The 2360–2332 cm^−1^ peaks of modified fly ash are weakened or even disappeared, while the 1610 cm^−1^ peak becomes sharper, indicating that the original C≡C and C≡N have been oxidized to C=O and N=O. This is mainly generated by the loss on ignition of fly ash, i.e., the unburned carbon. The peak shape of unmodified fly ash near 1100 cm^−1^ is sharp because it contains a lot of SiO_2_, which is combined as Si–O–Si, and the peak shape becomes broader and flatter after modification, indicating that the crystallinity of SiO_2_ is destroyed and becomes more dispersed. Overall, N_2_ modification is weaker than O_2_ modification in terms of increasing O–H and destroying SiO_2_ crystallinity.

Combining Figs. 6 and 7, it can be inferred that O_2_-modified fly ash can produce high-energy charged particles in a plasma reactor, which can more effectively oxidize the functional groups on fly ash surface, generating more active sites of C=O and N=O and thus promoting catalytic reactions^14^.

#### Study on the influence of parameter change

In the orthogonal experiments of 10 groups, BET and Boehm titration analyses were performed, and the results are shown in Table 6.

**Table 6:** Bet and Boehm titration results of fly ash.

No.	Denitration rate (%)	BET				Boehm		
		Specific surface area (m^2^ g^−1^)	Adsorption pore (nm)	Total pore volume (cm^3^ g^−1^)	Acid group (mmol g^−1^)	Basic group (mmol g^−1^)	Ph-OH (mmol g^−1^)	Carboxyl (mmol g^−1^)
1	38.88	1.383	15.993	0.0035	0.328	2.258	0.062	0.083
2	51.14	1.339	15.349	0.0040	0.195	2.358	0.002	0.061
3	51.23	1.298	12.449	0.0030	0.205	2.030	0.047	0.069
4	52.18	1.761	14.286	0.0033	0.119	2.228	0.021	0.090
5	53.23	1.172	13.756	0.0040	0.487	2.587	0.025	0.048
6	47.03	1.518	13.329	0.0034	0.279	2.253	0.051	0.072
7	50.25	1.71	14.433	0.0042	0.264	2.420	0.025	0.082
8	53.61	1.677	12.902	0.0034	0.658	2.775	0.032	0.031
9	49.81	1.947	12.333	0.0029	0.475	2.530	0.055	0.045
10	42.83	1.506	14.575	0.0035	0.393	2.309	0.061	0.069

Using specific surface area, adsorption pore size, total pore volume, acidic groups, basic group, phenolic hydroxyl groups, and carboxyl groups as independent variables and denitration rate as the dependent variable, linear regression analysis was performed. The VIF value in the obtained model is greater than 10, which means that there is a collinearity problem, and there are closely related independent variables in the model.

Generally, the mean pore size is proportional to the specific pore volume and inversely proportional to the specific surface area. Combining the *p*-value significance of adsorption pore size, the specific surface area,and total pore volume were removed as independent variables. Acidic groups include phenolic hydroxyl and carboxyl groups, carboxyl groups decompose at temperatures ranging from 200 to 300 °C, but the content is too low. Phenolic hydroxyl groups have a high content but usually decompose at temperatures higher than 600 °C^15^. Therefore, hydroxyl and carboxyl groups were removed as independent variables. By refitting the model, Table 7 can be obtained.

**Table 7:** Linear regression analysis results of 3 independent variables.

	Normalization coefficient	*t*	*p*	VIF	*R*^2^	*F*
Constant	–	1.890	0.108	–	0.789	*F* (3,6) = 7.487
Adsorption pore	− 0.758	− 3.685	0.010*	1.205		
Acid group	− 1.262	− 3.388	0.015*	3.946		
						*p* = 0.019
Basic group	1.295	3.657	0.011*	3.568		

*p < 0.05, *DW* = 2.206.

The model’s *R*^2^ value of 0.789 indicates that the adsorption pore size, acidic groups, and basic group can explain 78.9% change of the variation in denitration rate. The model passed the F-test, and all the VIF values were less than 5, indicating that there is no collinearity problem. The *DW* value is around 2, indicating that the model does not exhibit autocorrelation and that there is no correlation between the sample data, indicating a good model.

The model shows that the basic group have a significant positive effect on denitration rate, while the adsorption pore size and acidic groups have a significant negative effect on denitration rate. This suggests that the more basic group generated by plasma modification, the more denitration rate can be increased, while an increase in acidic groups will actually reduce denitration rate. Meanwhile, the smaller the adsorption pore size, the easier it is to improve the denitration rate.

The variance analysis of the orthogonal experimental results shows that the input power and discharge gap have a significant effect on denitration rate. Examining the effects of these two factors on adsorption pore size and basic group, as shown in Tables 8 and 9.

**Table 8:** Variance analysis of adsorption pore size.

	Sum of squares	d*f*	Mean square	*F*	*p*
Model	0.3961	4	0.099	3.93	0.0828**
Input power	0.1028	2	0.0514	2.04	0.2249
Discharge gap	0.3228	2	0.1614	6.41	0.0417*
Residual	0.1259	5	0.0252		

*p < 0.05, **p < 0.10, *R*^2^ = 0.7588.

**Table 9:** Variance analysis of basic group.

	Sum of squares	d*f*	Mean square	*F*	*p*
Model	0.2026	4	0.0506	6.5	0.0323*
Input power	0.1937	2	0.0969	12.44	0.0115*
Discharge gap	0.0114	2	0.0057	0.7345	0.5252
Residual	0.0389	5	0.0078		

*p < 0.05, *R*^2^ = 0.8388.

In Table 8, the model is significant at the 0.10 level. From the *p*-value observation, the discharge gap has a significant effect on adsorption pore size. Therefore, reducing the discharge gap can simultaneously reduce the adsorption pore size. In Table 9, the model is significant at the 0.05 level. From the *p*-value observation, the input power has a significant effect on the basic group. Therefore, increasing the input power can simultaneously increase the number of basic groups.

## Discuss

**Figure 9:**
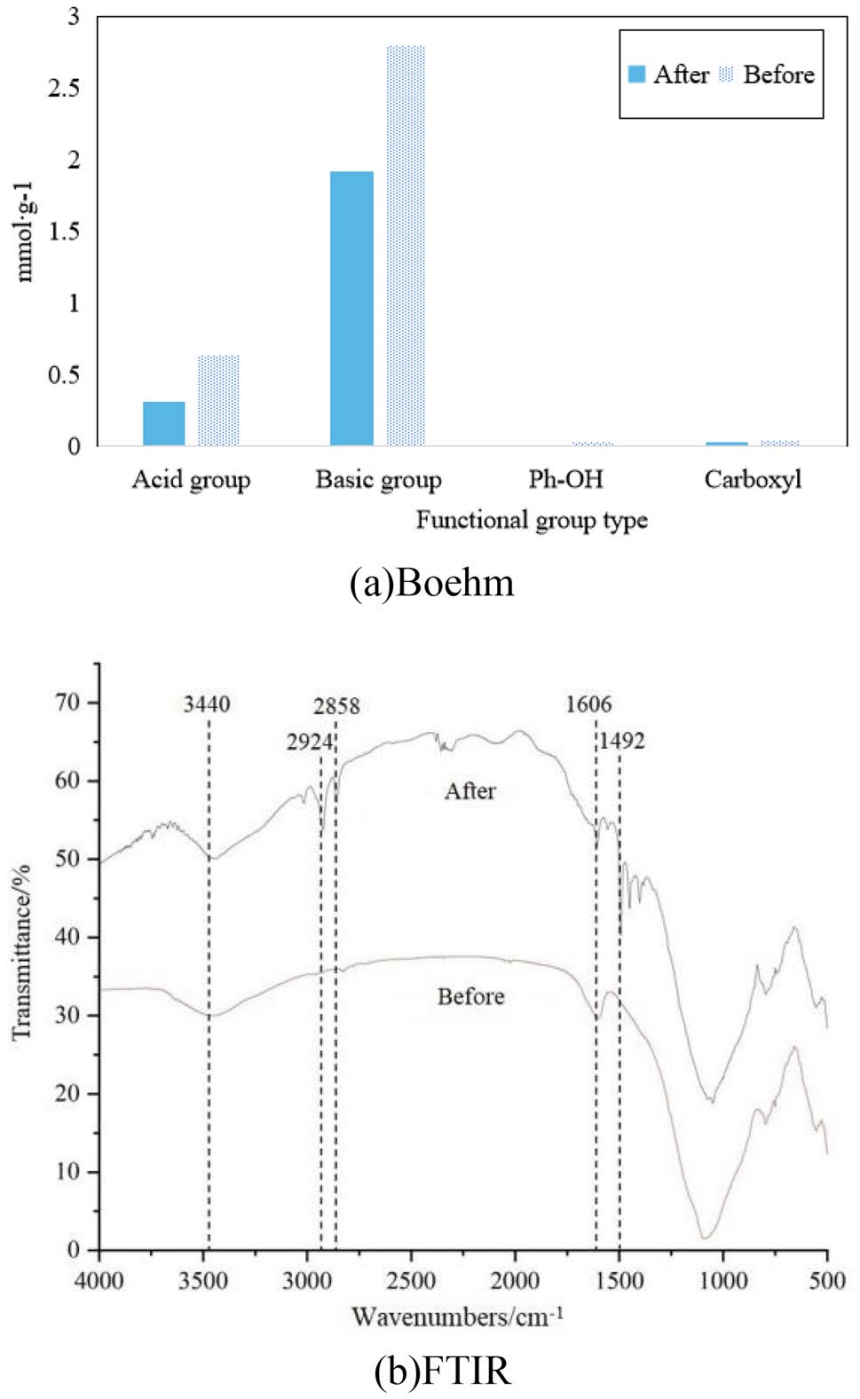
Functional group changes of fly ash before and after denitration.

The functional group changes of fly ash before and after denitration were characterized by Boehm titration and FTIR analysis, as shown in Fig. 9.

In Fig. 9a, the number of surface functional groups of the modified fly ash all decreased after reaction, and the reduction in basic functional groups was greater than that of acidic group.

In Fig. 9b, both the composition and content of surface functional groups of fly ash changed significantly after denitration. The peak at 3440 cm^−1^ was attributed to the vibration of O–H, and it was inferred that crystalline water was physically adsorbed on the surface after the loss of oxygen free radicals, based on the actual engine operating conditions. The peaks at 2924 cm^−1^ and 2858 cm^−1^ were both related to the vibration of CH_2_, indicating that residual hydrocarbons in the engine exhaust are adsorbed on the surface of fly ash catalyst. The continuous stretching vibration near 1492 cm^−1^ was due to the unburned alkane in diesel fuel. The peak at 1606 cm^−1^ was attributed to the characteristic peaks of C=O, C=N, and N=O, and its decrease after denitration indicated the involvement of the corresponding functional groups in chemical reactions. Therefore, C=O, C=N, and N=O were found to have a positive impact on the catalytic effect of the SCR reaction.

**Figure 10:**
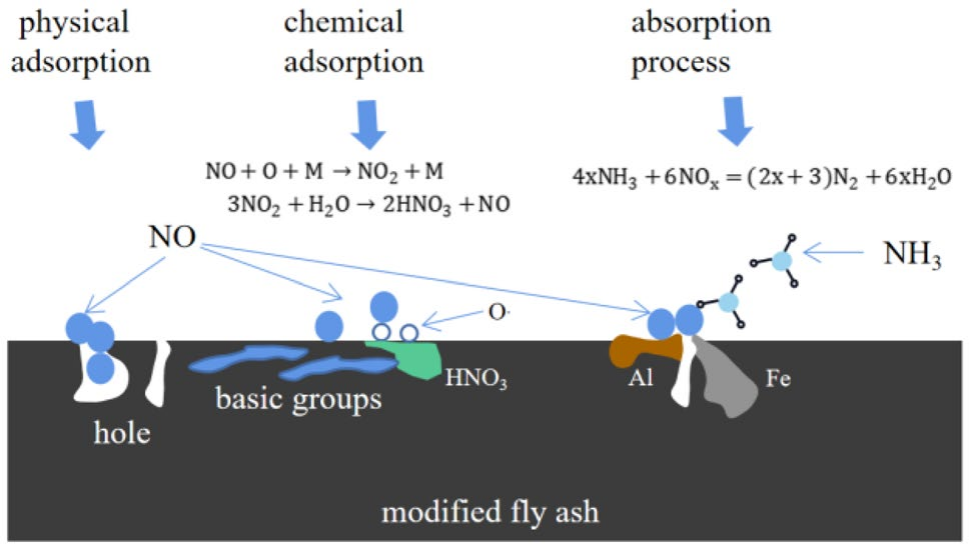
Mechanism of denitration of modified fly ash.

It is speculated that the possible denitration mechanism of plasma-modified fly ash includes physical adsorption, chemical adsorption, and absorption processes, as shown in Fig. 10.

Due to its porous structure and large surface area, NO and NO_2_ molecules are enriched on the surface through physical adsorption^16^. And the smaller the surface pore size, the more favorable it is for adsorption.

Some NO also reacts with surface oxygen free radicals to generate NO_2_^17^, as shown in Eq. (2).

2 $$ {\text{NO}} + {\text{O}} + {\text{M}} \to {\text{NO}}_{2} + {\text{M}} $$

Here, M is a third-party substance similar to N2. Some of the NO_2_ reacts with water on the surface to produce acid^18^, as shown in Eq. (3).

3 $$ 3{\text{NO}}_{2} + {\text{H}}_{2} {\text{O}} \to 2{\text{HNO}}_{3} + {\text{NO}} $$

In the low-temperature plasma reactor, basic group are generated on the surface of fly ash. Chemical adsorption occurs when basic group react with acid. The presence of acidic group weakens this adsorption process, resulting in a negative impact on denitration.

At the same time, the plasma modification treatment results in etching effects that disperse the active components, enhance the activity intensity of active centers, and expose more active sites^19^. The Fe and Al oxides exposed in fly ash produce mutual interactions and synergistic effects between metal carriers. Under their joint catalysis^20^, NO_x_ reacts with NH_3_, resulting in the absorption of NO_x_, as shown in Eq. (4).

4 $$ 4{\text{xNH}}_{3} + 6{\text{NO}}_{{\text{x}}} = \left( {2{\text{x}} + 3} \right){\text{N}}_{2} + 6{\text{xH}}_{2} {\text{O}} $$

The adsorption pore size explains the physical adsorption mechanism, while the alkaline groups explain the chemical adsorption mechanism. (In fact, this mechanism also includes the role of acidic groups, but the effects of acidic groups are negative, which will be offset by the effects of alkaline groups. Therefore, only the representation with alkaline groups was used.) In order to obtain the proportions of different mechanisms, the absolute values of the standardized coefficients in Table 7 were calculated and then divided by their sum (3.315). Among them, the proportion of the adsorption pore size is 0.2287, indicating that the impact of the adsorption pore size on the denitrification rate accounts for 22.87%. The remaining proportion of alkaline groups (acidic groups) is 0.7713, indicating that the influence of alkaline groups (acidic groups) on the denitrification rate accounts for 77.13%. Since the linear regression analysis model can only explain 78.9% of the variance, these proportions need to be multiplied by 0.789, yielding values of 18.04 and 60.86, indicating that 18.04% of the variance explained by the 78.9% is due to physical mechanisms, while 60.86% is due to chemical mechanisms. In addition, the unexplained variation of denitrification rate beyond 78.9%, i.e., 100–78.9% = 21.1%, can be attributed to the absorption process.

## Conclusions

1. Plasma modification treatment did not change the main mineral composition of fly ash, but only caused etching on the surface and reduced the adsorption pore size. The modification effect under an oxygen atmosphere was better than that under a nitrogen atmosphere.
2. The order of the influence of different plasma modification factors on denitration rate of fly ash was as follows: input power > discharge gap > discharge length > ionization time, and input power and discharge gap had a significant effect on denitration rate. The optimal combination of modification parameters was an input power of 40 W, a discharge gap of 2 mm, an ionization time of 30 min, and a discharge length of 80 mm.
3. Plasma modification of fly ash under an oxygen atmosphere mainly increased denitration rate by increasing basic group and reducing the adsorption pore size. Meanwhile, an increase in acidic groups had a significant negative impact. Reducing the discharge gap could reduce the adsorption pore size of fly ash. Increasing input power can increase the number of alkaline groups on the surface of fly ash.
4. The denitration mechanism of plasma-modified fly ash includes physical adsorption, chemical adsorption, and absorption processes. Among them, chemical adsorption plays a major role, accounting for about 60.86% of the denitrification rate.
